# Urinary excretion of RAS, BMP, and WNT pathway components in diabetic kidney disease

**DOI:** 10.14814/phy2.12010

**Published:** 2014-05-11

**Authors:** Maryam Afkarian, Irl B. Hirsch, Katherine R. Tuttle, Carla Greenbaum, Jonathan Himmelfarb, Ian H. de Boer

**Affiliations:** 1 Kidney Research Institute and Division of Nephrology, Department of Medicine, University of Washington, Seattle, Washington; 2 Division of Metabolism, Endocrinology and Nutrition, Department of Medicine, University of Washington, Seattle, Washington; 3 Providence Sacred Heart Medical Center, Spokane, Washington; 4 Diabetes Research Program, Benaroya Institute, Seattle, Washington; 5 Department of Epidemiology, University of Washington, Seattle, Washington

**Keywords:** BMP pathway, diabetic kidney disease, pathophysiology, renin–angiotensin system, WNT pathway

## Abstract

The renin–angiotensin system (RAS), bone morphogenetic protein (BMP), and WNT pathways are involved in pathogenesis of diabetic kidney disease (DKD). This study characterized assays for urinary angiotensinogen (AGT), gremlin‐1, and matrix metalloproteinase 7 (MMP‐7), components of the RAS, BMP, and WNT pathways and examined their excretion in DKD. We measured urine AGT, gremlin‐1, and MMP‐7 in individuals with type 1 diabetes and prevalent DKD (*n* = 20) or longstanding (*n* = 61) or new‐onset (*n* = 10) type 1 diabetes without DKD. These urine proteins were also quantified in type 2 DKD (*n* = 11) before and after treatment with candesartan. The utilized immunoassays had comparable inter‐ and intra‐assay and intraindividual variation to assays used for urine albumin. Median (IQR) urine AGT concentrations were 226.0 (82.1, 550.3) and 13.0 (7.8, 20.0) *μ*g/g creatinine in type 1 diabetes with and without DKD, respectively (*P* < 0.001). Median (IQR) urine gremlin‐1 concentrations were 48.6 (14.2, 254.1) and 3.6 (1.7, 5.5) *μ*g/g, respectively (*P* < 0.001). Median (IQR) urine MMP‐7 concentrations were 6.0 (3.8, 10.5) and 1.0 (0.4, 2.9) *μ*g/g creatinine, respectively (*P* < 0.001). Treatment with candesartan was associated with a reduction in median (IQR) urine AGT/creatinine from 23.5 (1.6, 105.1) to 2.0 (1.4, 13.7) *μ*g/g, which did not reach statistical significance. Urine gremlin‐1 and MMP‐7 excretion did not decrease with candesartan. In conclusion, DKD is characterized by markedly elevated urine AGT, MMP‐7, and gremlin‐1. AGT decreased in response to RAS inhibition, suggesting that this marker reflects therapeutic response. Urinary components of the RAS, BMP, and WNT pathways may identify risk of DKD and aid development of novel therapeutics.

## Abstract (precis)

Urine angiotensinogen, matrix metalloproteinase‐7, and gremlin‐1 concentrations are markedly elevated in people with type 1 diabetes and kidney disease, compared with those with recently diagnosed type 1 diabetes or longstanding type 1 diabetes without kidney disease. Treatment with an inhibitor of the renin–angiotensin system tended to reduce urine angiotensinogen concentration, but not urine matrix metalloproteinase‐7 or gremlin‐1.

## Introduction

Diabetic kidney disease (DKD) affects 20–40% of individuals with diabetes (Krolewski et al. 1985; Nathan et al. 2009; de Boer et al. 2011a,b). Moreover, its presence and severity is a strong predictor of long‐term mortality (Groop et al. 2009; Orchard et al. 2010; Afkarian et al. 2013). Development of novel diagnostic and therapeutic tools is dependent on a deeper understanding of underlying pathophysiologic mechanisms.

Evidence from animal models of DKD supports contribution of three pathways to DKD development: the renin–angiotensin system (RAS), WNT, and bone morphogenetic protein (BMP) pathways. Activation of the RAS pathway has been heavily implicated in DKD pathophysiology and inhibiting it has yielded effective therapeutic interventions currently in use (RAS inhibitors). Angiotensinogen (AGT) is the sole substrate for renin, which catalyzes the rate‐limiting step in the RAS pathway, eventually yielding angiotensin II, which mediates most of the distal effects of the RAS pathway. The BMP pathway activation counteracts TGF‐*β* signaling while BMP pathway antagonists, such as gremlin‐1, prevent BMP inhibition of TGF‐*β* signaling (Zeisberg et al. 2003). The WNT pathway activity, induced by TGF‐*β* signaling, is also increased in DKD and urinary excretion of MMP‐7 (matrix metalloproteinase 7), a target gene highly upregulated by WNT pathway activation, is shown to correlate with renal WNT pathway activity in animal models of kidney disease (He et al. 2012).

In this study, we sought to determine if urinary excretion of AGT, gremlin‐1, and MMP‐7 increases with activation of the RAS and WNT and inhibition of BMP pathways in DKD. To do so, we characterized intra‐ and interassay and intraindividual variability in the commercially available immunoassays and used these to compare urinary concentrations of AGT, MMP‐7, BMP‐7, and gremlin‐1 in people with type 1 diabetes and DKD to those without kidney disease as well as those with new‐onset type 1 diabetes. Additionally, we examined the change in urinary concentration of these pathway components in response to RAS pathway inhibition in type 2 diabetes.

## Material and Methods

### Study populations

Samples and clinical data from people with type 1 diabetes were obtained after informed consent either from the Kidney Research Institute Diabetic Kidney Disease Repository of the University of Washington or the Benaroya Research Institute Diabetes Translational Research Project. DKD was defined as either a urine albumin to creatinine ratio (ACR) ≥300 mg/g or an estimated glomerular filtration rate (GFR) <60 mL/min per 1.73 m^2^ and ACR ≥30 mg/g. People with longstanding diabetes but no evidence of DKD had ≥30 years of type 1 diabetes, estimated GFR >90 mL/min per 1.73 m^2^, and ACR <300 mg/g. New‐onset type 1 diabetes was defined as diagnosis of diabetes ≤12 months prior to urine sample collection with no hypertension or kidney disease. The use of these samples and data were approved by the Institutional Review Boards at the University of Washington and Benaroya Research Institute.

Samples and clinical data from people with type 2 diabetes receiving candesartan were obtained from an open label dose escalation study of candesartan cilexetil (Saha et al. 2010). This study included 11 participants with type 2 diabetes and DKD, defined as urine protein excretion >500 mg/day and 10 participants with type 2 diabetes and no DKD, defined as urine albumin excretion <30 mg/day. Exclusion criteria included patients with conditions associated with elevated TGF‐*β*1 levels, uncontrolled hypertension, creatinine clearance <30 mL/min, serum creatinine >3 mg/dL, hemoglobin A1c >10% or major comorbidities. After a 2‐week washout, the participants with DKD received candesartan doses escalating from 8 to 64 mg/day over a 12‐week follow‐up period. Use of the deidentified stored samples and participant data from this study was considered exempt from review by the Human Subjects Division of the University of Washington.

### Sample collection and storage

For samples from the population with type 1 diabetes, a spot clean‐catch midstream urine sample was obtained and stored at 4°C immediately after collection until processed. To process, urine samples were centrifuged at 4700*g* for 15 min at 4°C and supernatant was aliquoted and stored at −80°C until further use. The mean (standard deviation, SD) time from collection to −80°C storage was 5.7 (2) h. For the samples from the population with type 2 diabetes, 24‐h urine was collected at baseline and prior to administration of the daily candesartan dose at the end of each 3‐week dose escalation cycle and stored at −70°C until subsequent use (Saha et al. 2010). For this study, urine samples from the baseline (no candesartan), middle (candesartan 16 mg/day), and end of the study (candesartan 64 mg/day) were used.

### Laboratory measurements

Urine samples were thawed at 37°C for 3 min, vortexed, and either used directly for immunoassays (MMP‐7, AGT) or concentrated fivefold (gremlin‐1) using Amicon 10 kDa ultrafiltration units (Millipore, Billerica, MA) following manufacturer instructions. Urine AGT was measured using a quantitative solid‐phase sandwich enzyme‐Linked immunosorbent assay (ELISA) distributed by IBL‐America (Minneapolis, MN), with a minimum detection limit of 30 pg/mL and <0.1% cross‐reactivity with human angiotensin I, II, III, or IV, angiotensin (1–9), or angiotensin (1–7). Urine gremlin‐1 was measured using a quantitative solid‐phase sandwich ELISA (Uscnk Life Sciences, Wuhan, China), with a minimum detection limit of 60 pg/mL and no significant cross‐reactivity with Cerberus, CTGF, BDNF, or CYR61. Urine MMP‐7 was measured using a quantitative solid‐phase sandwich ELISA (R&D Systems, Minneapolis, MN) with a minimum detection limit of 20 pg/mL and no significant cross‐reactivity with other human MMP or TIMPs.

### Assay characterization

Inter‐ and intra‐assay variability was determined as the coefficient of variation (CV) in technical replicates of the same samples tested on the same day (intra‐assay) or different days (interassay). For intraindividual variation, CVs were calculated from measurements in spot and 24‐h urine samples, collected from the 11 individuals with type 2 diabetes and DKD over a 12‐month interval. To determine protein stability at room temperature, urine samples, collected from six individuals with type 2 diabetes and DKD, were incubated at room temperature from 1 to 48 h. To evaluate the effect of frequent freeze–thaw, urine samples obtained from six individuals with type 2 diabetes and DKD underwent one, two or five consecutive freeze–thaw cycles in liquid nitrogen prior to protein quantification.

### Statistical analysis

Data are represented as mean (SD) for normally distributed data and median (interquartile range, IQR) for non‐normal data. Non‐normal data were compared using the Mann–Whitney test. We used multiple linear regression analysis to test differences in urine proteins, adjusting for age, gender, race, and diabetes duration. *P*‐values less than 0.05 were considered statistically significant. Analyses were performed using the SAS 9.3 statistical software package (Carey, NC).

## Results

### Assay and analyte characterization

**Figure 1.**
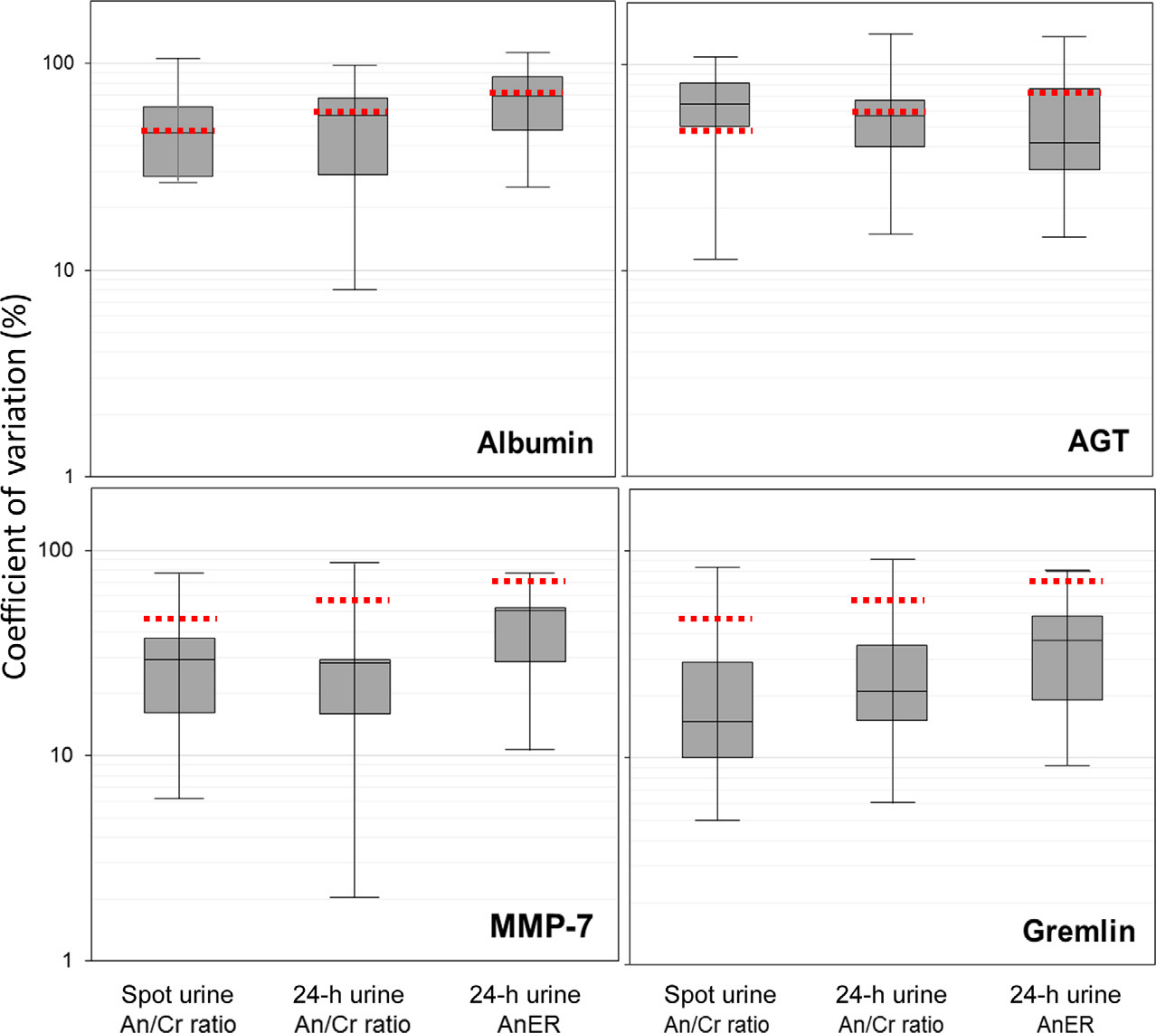
Intraindividual variability in urine AGT, gremlin, and MMP‐7. Distribution of coefficients of variation (CV) for each analyte measured in urine samples collected at 0, 3, and 12 months. An/Cr, analyte to creatinine ratio in spot and 24‐h urine collections; AnER, analyte excretion rate. The dashed lines represent the intraindividual CVs for urine albumin excretion in the same samples.

Gremlin‐1 concentrations in unconcentrated urine were too low to reliably detect. Urine concentration using ultrafiltration was selected from among four tested concentration methods because of a combination of ease, high yield, and low variability (data not shown). We characterized the commercially available assays for AGT, gremlin‐1, and MMP‐7 for intra‐ and interassay as well as intraindividual variation, protein stability after room temperature incubation, and repeated freeze–thaws (Table****1). The mean intra‐ and interassay variation was ≤20% for all proteins, highest for gremlin‐1 and lowest for MMP‐7. Intraindividual variation over a 12‐month interval for AGT was comparable to that of albumin and for gremlin‐1 and MMP‐7 was lower than that of albumin. Urine MMP‐7 and gremlin‐1 had lower variation overtime in spot samples than 24‐h samples. Urine AGT had somewhat higher variation in spot samples than 24 h samples overtime (Table 1 and Fig. 1). After a 6‐h incubation at room temperature, AGT, gremlin‐1, and MMP‐7 were all stable (89%, 93%, and 99% of reference, respectively). However, after a 24‐h incubation, urine AGT and gremlin‐1 dropped significantly (68% and 86% of reference, respectively) while MMP‐7 was more stable (94% of reference). Urine AGT and MMP‐7 were minimally reduced after five freeze–thaw cycles, while urine gremlin‐1 concentration dropped 12% between the second and fifth freeze–thaw (99% to 87%) (Table****1). Furthermore, addition of protease inhibitors did not affect urine AGT, gremlin‐1, and MMP‐7 concentration (data not shown).

**Table 1.** Characterization of the immunoassays for angiotensinogen, gremlin‐1, and MMP‐7.

	Albumin	Angiotensinogen	Gremlin‐1	MMP‐7
Intra‐assay CV (%)	ND	8.2 (5.4)	13.3 (4.4)	5.4 (5.2)
Interassay CV (%)	ND	14 (9)	20 (14)	9 (7)
Intraindividual CV over 12 months (%)				
24 h AER	64	42	37	51
24 h ACR	56	57	21	28
Spot ACR	46	64	15	29
Stability at room temperature (% of reference)				
1 h incubation (reference)	ND	100	100	100
6 h incubation		89	93	99
24 h incubation		68	86	94
48 h incubation		63	51	88
Stability to freeze–thaw (% of reference)				
1 freeze–thaw (reference)	ND	100	100	100
2 freeze–thaws		95	99	98
5 freeze–thaws		92	87	94

Values for intra‐ and interassay CVs are represented as mean (SD). ND, not determined.

### Study populations

Individuals with DKD were younger than those with longstanding type 1 diabetes but older than those with new‐onset diabetes, as expected (Table****2). They also included fewer women (30% compared with 56% and 60% in groups with longstanding and new‐onset diabetes, respectively) and fewer Caucasians (85% vs. 97% and 100% in groups with longstanding and new‐onset diabetes, respectively). Mean (SD) diabetes duration was 28 (7) years in the group with DKD versus 39 (6) years in those with longstanding diabetes. Mean hemoglobin A1c levels were lower in the group with longstanding diabetes without DKD than the one with DKD (7.4 vs. 8.7). Hemoglobin A1c levels were not available for the subgroup with new‐onset diabetes. As expected from selection criteria, mean GFR was much lower in the group with DKD than the control populations with longstanding or new‐onset type 1 diabetes and no DKD (49, 93, and 120 mL/min per 1.73 m^2^, respectively). For the same reason, mean ACR exceeded 300 mg/g in people with DKD, but was normal (<30 mg/g) in both control populations (Table****2). The population with type 2 diabetes has been previously described in detail (Saha et al. 2010).

**Table 2.** Characteristics of the participants with type 1 diabetes.

	Longstanding diabetes with no kidney disease^1^	Diabetic kidney disease^1^	New‐onset diabetes with no kidney disease^1^	*P*‐value
	Mean (SD) or number (proportion, %)	Mean (SD) or number (proportion, %)	Mean (SD) or number (proportion, %)	
*N*	61	20	10	
Age (years)	50 (9)	43 (9)	30 (14)	<0.001
Female *N* (%)	34 (56)	6 (30)	6 (60)	0.11
Caucasian *N* (%)	59 (97)	17 (85)	10 (100)	0.31
DM duration (Years)	39 (6)	28 (7)	1 (0)	<0.001
Hemoglobin A1c (%)	7.4 (0.8)	8.7 (1.2)	NA	<0.001
GFR (mL/min per 1.73 m^2^)	93 (12)	49 (24)	120 (24)	<0.001
ACR (mg/g)	10 (11)	680 (588)	7 (4)	<0.001
RAAS inhibitor use (%)	52	90	0	<0.001

^1^ DKD was defined as a urine albumin to creatinine ratio (ACR) ≥300 mg/g or estimated GFR <60 mL/min per 1.73 m^2^ and ACR ≥30 mg/g. Longstanding diabetes with no kidney disease were individuals with ≥30 years of type 1 diabetes, estimated GFR >90 mL/min per 1.73 m^2^, and ACR <300 mg/g. NA, hemoglobin A1c levels were not available for the subgroup with new‐onset diabetes.

### Urinary concentration of AGT, MMP‐7, and gremlin‐1 in type 1 diabetes

**Figure 2.**
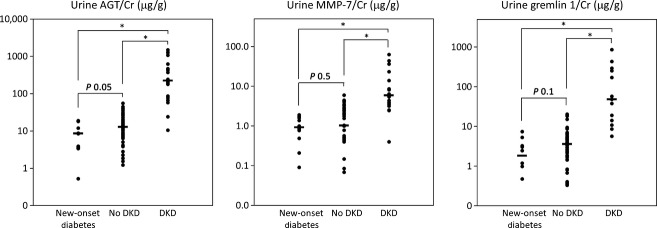
Urine AGT, MMP‐7, and gremlin‐1 in type 1 diabetes with or without DKD. Urine AGT/Cr, MMP‐7/Cr, and gremlin‐1/Cr in random urine samples from people with new‐onset T1D, longstanding T1D with no DKD, and people with T1D and DKD. *P*‐values are from Mann–Whitney comparison between the data‐points. *P < 0.001.

Urine concentration of AGT, MMP‐7, and gremlin‐1 showed statistically significant moderate correlations with albuminuria in the combined cohort (*R*^*2*^ = 0.54, 0.41, and 0.20 for AGT, MMP‐7, and gremlin‐1, respectively; *P* < 0.001) (Table****3). Creatinine‐adjusted urine AGT was 17‐fold higher with DKD than without DKD and 26‐fold higher than urine AGT concentration at the onset of type 1 diabetes (Fig. 2). Median urine AGT/Cr (AGT concentration normalized to urine creatinine) in cases (226.0 ng/mg) was significantly different from those in controls (13.0 ng/mg) and participants with new‐onset type 1 diabetes (8.7 ng/mg). These relations were unaffected by adjustment for age, race, gender, diabetes duration, and RAS inhibitor use, as well as correction for multiple testing using the Bonferroni method (*P* < 0.001). The difference in median AGT/Cr levels between people with DKD versus longstanding diabetes without DKD remained significant after additional adjustment for hemoglobin A1c (*P* < 0.001).

**Table 3.** Correlations (*R*^2^ linear regression) between urinary excretion of albumin, AGT, gremlin‐1, and MMP‐7.

	ACR	MMP7	AGT	Gremlin‐1
ACR	1.00	0.41	0.54	0.20
MMP7		1.00	0.25	0.07
AGT			1.00	0.09
Gremlin‐1				1.00

All correlations are statistically significant (*P* < 0.001).

Creatinine‐adjusted urine MMP7 concentration was sixfold higher in people with type 1 diabetes and DKD than those with no DKD and 6.7‐fold higher than in newly diagnosed diabetes. Median urine MMP‐7/Cr (MMP‐7 concentration normalized to urine creatinine) in cases (6.0 ng/mg) was significantly different from those in controls (1.0 ng/mg) and participants with new‐onset type 1 diabetes (0.9 ng/mg). These relations were unaffected by adjustment for age, race, gender, diabetes duration, and RAS inhibitor use, as well as correction for multiple testing using the Bonferroni method (*P* < 0.001) (Fig. 2). The difference in median MMP‐7/Cr levels between people with DKD versus longstanding diabetes without DKD remained significant after additional adjustment for hemoglobin A1c (*P* < 0.001).

Creatinine‐adjusted urine gremlin‐1 concentration was 13‐fold higher in people with type 1 diabetes and DKD than those with no DKD and 27‐times higher than urine gremlin‐1 concentration at the onset of type 1 diabetes. Median urine gremlin‐1/Cr (gremlin‐1 concentration normalized to urine creatinine) in cases (48.6 ng/mg) was significantly different from those in controls (3.6 ng/mg) and participants with new‐onset type 1 diabetes (1.8 ng/mg) (Fig. 2). These relations were unaffected by adjustment for age, race, gender, diabetes duration, and RAS inhibitor use, as well as correction for multiple testing using the Bonferroni method (*P* < 0.001). The difference in median gremlin‐1/Cr levels between people with DKD versus longstanding diabetes without DKD remained significant after additional adjustment for hemoglobin A1c (*P* < 0.001). USAG1, another BMP pathway antagonist, was present in urine in concentrations 1000‐fold lower than gremlin‐1 and was not further pursued. Urine BMP‐7 concentration was not detectable in samples concentrated up to 200‐fold and as such was estimated to be less than 0.5 pg/mL.

### Effect of RAS inhibition on urine concentration of AGT, MMP‐7, and gremlin‐1 in type 2 diabetes

**Figure 3.**
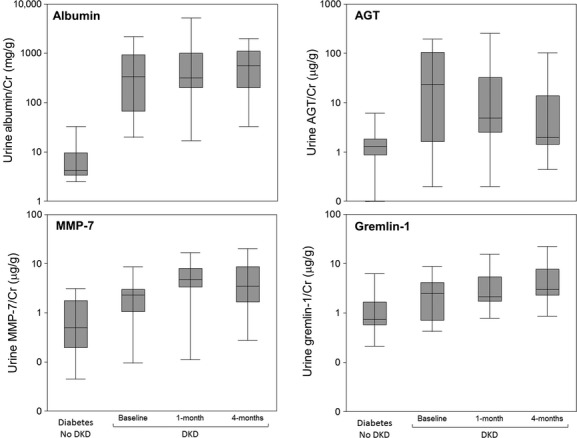
Urine AGT, gremlin‐1, and MMP‐7 before and after treatment with candesartan in type 2 diabetes. Urine AGT concentration is lower after 4.2 (1.7) months of candesartan. Urine MMP‐7 and gremlin‐1 are not altered by candesartan. These differences were not statistically significant, likely due to small sample sizes. The 1‐month visit occurred 1.1 (1.3) months after baseline visit; the 4‐month visit occurred 4.2 (1.7) months after the baseline visit. Proteins were measured in 24 h urine collections.

Median (IQR) of urine AGT/Cr concentration was 23.5 (1.6, 105.1) at study baseline (before candesartan) and 2.0 (1.4, 13.7) after 4 months of treatment with candesartan (candesartan 64 mg/day at study end). Median (IQR) urine MMP‐7 were 2.3 (1.1, 3.0) and 3.5 (1.7, 8.7) at study baseline and end. Median (IQR) urine gremlin‐1 were 2.5 (0.7, 4.2) and 3.0 (2.3, 7.8) at study baseline and end (Fig. ****3). Of the 11 participants with DKD, six had lower albuminuria at the end of the study (“responders”), while five had equal or higher albumin concentration at the end of the study (“nonresponders”). Median (IQR) urine AGT/Cr tended to be higher in responders than nonresponders (67.4 (25.6, 118.8) and 1.3 (1.1, 2.2), respectively). Median (IQR) urine gremlin‐1 was 3.6 (2.7, 4.3) in responders versus 0.5 (0.5, 1.5) in nonresponders. Median (IQR) urine MMP‐7 concentration was 3.3 (2.7, 4.0) in nonresponders versus 1.8 (0.9, 2.3) in responders. These differences were not statistically significant, likely due to small sample sizes.

## Discussion

The primary finding of this study is that urine AGT, MMP‐7, and gremlin‐1 concentration are markedly elevated in people with type 1 diabetes and DKD, compared with those with recently diagnosed type 1 diabetes or those with longstanding type 1 diabetes without DKD. We also found that in type 2 diabetes, treatment with an angiotensin receptor antagonist tended to reduce urine AGT concentration, but not urine MMP‐7 or gremlin‐1. Urine AGT, MMP‐7, and gremlin‐1 were quantified using optimized commercially available assays, which had acceptable inter‐ and intra‐assay variation, comparable or better intraindividual variability than that of albumin, and stable protein quantification after up to 6 h at room temperature or two freeze–thaws.

Data from animal models and some human studies support involvement of the RAS, BMP, and WNT pathways in DKD pathophysiology. Kidney AGT expression is increased in several DKD animal models, where AGT is induced by angiotensin II and promotes progression of kidney disease (Kobori et al. 2002, 2003; Singh et al. 2005; Liu et al. 2007; Ohashi et al. 2010). AGT expression is also increased in human biopsies with DKD (Lai et al. 1998) and urine samples from people with type 2 diabetes and kidney disease (Kobori et al. 2008; Kim et al. 2012). Furthermore, urinary AGT concentration strongly correlates with renal angiotensin II and RAS pathway activity (Yamamoto et al. 2007; Nishiyama et al. 2011), suggesting that AGT may be both a biologic mediator and a read‐out of the RAS activity. Urine AGT is associated with GFR loss over 2 years of follow‐up in type 2 diabetes, even after adjustment for baseline proteinuria and GFR (Yamamoto et al. 2007). Compared with individuals without diabetes, urine AGT concentration is also increased in people with type 1 diabetes and normal urine albumin excretion (Saito et al. 2009). However, urine AGT concentration has not been reported in type 1 diabetes with kidney disease.

The central mediator of the WNT pathway, *β*‐catenin, is activated (translocated to the nuclei) in kidneys of several DKD animal models (Kato et al. 2011; Rooney et al. 2011; Zhou et al. 2012) and inhibition of the WNT pathway ameliorates podocyte injury, albuminuria, and fibrosis (Dai et al. 2009; He et al. 2009, 2011; Hao et al. 2011), though one report suggests a more complex picture with some WNT pathway activity necessary for podocyte survival (Kato et al. 2011). Increased expression of WNT pathway components has also been noted in human DKD biopsies (Cohen et al. 2008; Dai et al. 2009; Kato et al. 2011; He et al. 2012), with MMP‐7 being the most highly induced WNT target in mRNA arrays of diabetic kidney tissue (Cohen et al. 2008). While urine MMP‐7 correlates with WNT pathway activity in animal models (He et al. 2012), urine MMP‐7 concentration has not been studied in humans.

In animal models of DKD, kidney expression of BMP‐7 is reduced and gremlin‐1 is increased long before the onset of overt DKD (McMahon et al. 2000; Wang et al. 2001; Yeh et al. 2009). Inhibition of the BMP pathway by reducing BMP‐7 expression leads to mesangial matrix expansion (Miyazaki et al. 2006), while activation of the pathway by reducing gremlin‐1 or overexpressing BMP‐7 expression ameliorates DKD manifestations (Wang et al. 2003, 2006; Sugimoto et al. 2007; Roxburgh et al. 2009; Zhang et al. 2010). Human biopsies of DKD also demonstrate elevated gremlin‐1 and reduced BMP‐7 expression (Dolan et al. 2005; De Petris et al. 2007; Walsh et al. 2008; Turk et al. 2009). However, urine concentration of BMP pathway components has not been described in human kidney disease.

We find that urine concentration of AGT, MMP‐7, and gremlin‐1 is low at the onset of type 1 diabetes and remains low in people who have intact kidney function after 30 or more years of type 1 diabetes. However, the concentration of each protein is markedly elevated in people with type 1 diabetes who develop DKD. Examining urinary concentration of these proteins in people with intact kidney function (GFR >90 mL/min per 1.73 m^2^ and no macroalbuminuria) after 30 or more years of diabetes avoids the potential misclassification of individuals with cross‐sectional normoalbuminuria as controls, since this latter group may harbor individuals with early or nonalbuminuric DKD. Furthermore, the finding of low urinary concentration of AGT, gremlin‐1, and MMP‐7 in new‐onset type 1 diabetes establishes a starting point for the trajectory of concentration of these proteins in the time course of DKD. This suggests that concentration of these proteins rises in urine of people with type 1 diabetes who go on to develop DKD, but not in those who do not develop DKD, possibly reflecting the known derangement of intrarenal RAS, WNT, and BMP pathways with progression of DKD. This raises the possibility that the increase in urinary concentration of these proteins may occur early enough in DKD course to be of value for detection of earlier stages of DKD. The cross‐sectional data presented here support examining association of urinary concentration of these proteins at earlier time points with subsequent development of DKD in a longitudinal study.

We also find that treatment with an inhibitor of the RAS pathway shows a trend toward reducing urine AGT to creatinine concentration, as reported previously (Yamamoto et al. 2007; Ogawa et al. 2009; Nishiyama et al. 2011), but not urine MMP‐7 or gremlin‐1. The heterogeneity in response to RAAS inhibitors in terms of ACR reduction is well‐documented (Hellemons et al. 2011; Holtkamp et al. 2011). In our cohort of 11 people, six had lower albuminuria at the end of the study (“responders”), while five had no reduction in albumin concentration at the end of the study (“nonresponders”). We found that the response to RAS inhibition in terms of reduction in albuminuria was observed in people with higher baseline urine AGT, as was also noted in prior studies (Ogawa et al. 2009; Jang et al. 2012). These findings suggest two interesting possibilities. First, if urine concentrations of these proteins are a reflection of intrarenal activities of the corresponding pathways, the WNT and BMP pathways appear unaffected with RAS inhibition and could present potential therapeutic targets in addition to the RAS pathway. A peptide agonist of the BMP pathway is currently being tested and has shown promise in reversal of kidney injury and fibrosis in animal models (Sugimoto et al. 2012). Furthermore, elevated urinary concentration of these proteins may identify patients in whom dysregulation of the corresponding pathway contributes to DKD, allowing optimal targeting of the respective therapies to appropriate subsets of patients.

Strengths of this study include a first‐pass evaluation, as potential biomarkers, of novel urinary proteins representing promising candidate pathways, quantification of these urine proteins using a well characterized process from urine collection to assay, and use of a carefully ascertained extreme case–control population. The study has limitations. As DKD was not confirmed by a kidney biopsy, inclusion of other causes of kidney disease among our cases is possible. Other limitations are the cross‐sectional study design, small sample size, and lack of parallel quantification of protein concentrations in serum or kidney tissue.

In conclusion, using carefully characterized assays, we find that in people with type 1 diabetes who have DKD, urine concentration of AGT, MMP‐7, and gremlin‐1 are markedly higher than in individuals with new onset of diabetes or those with longstanding diabetes without DKD. We also find a decrease in urine AGT, but not MMP‐7 or gremlin‐1, in response to RAS inhibition in people with type 2 diabetes and DKD, potentially reflecting therapeutic response. Urinary components of the RAS, BMP, and WNT pathways may identify risk of DKD as well as aid in development of novel therapies.

## Conflict of Interest

None declared.
